# Emergency Department Visits for COVID-19 by Race and Ethnicity — 13 States, October–December 2020

**DOI:** 10.15585/mmwr.mm7015e3

**Published:** 2021-04-16

**Authors:** Amanda R. Smith, Jourdan DeVies, Elise Caruso, Lakshmi Radhakrishnan, Michael Sheppard, Zachary Stein, Renee M. Calanan, Kathleen P. Hartnett, Aaron Kite-Powell, Loren Rodgers, Jennifer Adjemian

**Affiliations:** ^1^Epidemic Intelligence Service, CDC; ^2^CDC COVID-19 Response Team; ^3^Division of Health Informatics and Surveillance, Center for Surveillance, Epidemiology, and Laboratory Services, CDC; ^4^Office of the Director, National Center for Emerging and Zoonotic Infectious Diseases, CDC.

Hispanic or Latino (Hispanic), non-Hispanic Black or African American (Black), and non-Hispanic American Indian or Alaska Native (AI/AN) persons have experienced disproportionately higher rates of hospitalization and death attributable to COVID-19 than have non-Hispanic White (White) persons (*1*–*4*). Emergency care data offer insight into COVID-19 incidence; however, differences in use of emergency department (ED) services for COVID-19 by racial and ethnic groups are not well understood. These data, most of which are recorded within 24 hours of the visit, might be an early indicator of changing patterns in disparities. Using ED visit data from 13 states obtained from the National Syndromic Surveillance Program (NSSP), CDC assessed the number of ED visits with a COVID-19 discharge diagnosis code per 100,000 population during October–December 2020 by age and race/ethnicity. Among 5,794,050 total ED visits during this period, 282,220 (4.9%) were for COVID-19. Racial/ethnic disparities in COVID-19 ED visit rates were observed across age groups. Compared with White persons, Hispanic, AI/AN, and Black persons had significantly more COVID-19–related ED visits overall (rate ratio [RR] range = 1.39–1.77) and in all age groups through age 74 years; compared with White persons aged ≥75 years, Hispanic and AI/AN persons also had more COVID-19–related ED visits (RR = 1.91 and 1.22, respectively). These differences in ED visit rates suggest ongoing racial/ethnic disparities in COVID-19 incidence and can be used to prioritize prevention resources, including COVID-19 vaccination, to reach disproportionately affected communities and reduce the need for emergency care for COVID-19.

NSSP data were used to assess ED visits with a COVID-19 diagnosis code* during October 1–December 31, 2020. NSSP receives ED visit records from 71% of hospitals in the United States. Data from 13 states (Connecticut, Illinois, Maryland, Massachusetts, Michigan, Nevada, New Mexico, Oregon, Utah, Vermont, Virginia, Washington, and Wisconsin) meeting the following data quality thresholds were included in the study: >85% of facilities in the state report data to NSSP, >85% of the ED visits had complete and valid discharge diagnosis codes, >85% of ED visits included race data, and >85% of ED visits included ethnicity data. Data from before October 1, 2020 did not consistently meet these thresholds across all 13 states. COVID-19 ED visits were categorized by patient race/ethnicity (Hispanic, non-Hispanic AI/AN, non-Hispanic Asian or Pacific Islander [A/PI], non-Hispanic Black, and non-Hispanic White) and age group (0–17, 18–29, 30–44, 45–64, 65–74, and ≥75 years).

Race/ethnicity–specific crude and age-stratified visit rates per 100,000 population were calculated using population denominators from the National Center for Health Statistics’ 2019 bridged-race postcensal population estimates (*5*). Visits with patient ethnicity identified as Hispanic or Latino were categorized as Hispanic or Latino, even if race data were missing. Visits with patient ethnicity identified as not Hispanic or Latino with complete race data were categorized into one of the non-Hispanic race categories. Visits with patient ethnicity data missing or visits with patient ethnicity identified as non-Hispanic/Latino but missing patient race data were not included in the analysis.^†^ Race/ethnicity–specific crude and age-stratified RR^§^ with 95% confidence intervals (CIs) were calculated as the rate of COVID-19 ED visits among a racial/ethnic group divided by the rate of COVID-19 ED visits among White persons. RRs with CIs excluding 1.0 were considered statistically significant, and nonoverlapping CIs were used to identify differences in RRs by age groups. All analyses were conducted using R software (version 4.0.4; The R Foundation). This activity was reviewed by CDC and was conducted consistent with applicable federal law and CDC policy.^¶^

Among ED visits from 13 states during October 1–December 31, 2020, Hispanic persons were more likely to seek ED care for COVID-19 than were White persons overall (crude RR = 1.77) (Table 1) and for each age group examined (RR range = 1.91–2.92) (Table 2). Likewise, AI/AN persons were more likely to seek ED care for COVID-19 than were White persons, both overall (crude RR = 1.71) and among each age group (RR range = 1.22–3.07) (Table 2). Overall, Black persons aged ≤74 were more likely to seek ED care for COVID-19 compared with White persons (crude RR = 1.39) (Table 1), (age-stratified RR range = 1.54–2.19) (Table 2), but no differences between Black persons and White persons aged ≥75 years were observed. Fewer A/PI persons sought ED care for COVID-19 than did White persons overall (crude RR = 0.70) (Table 1) and among age groups ≤44 years and ≥75 years (RR range = 0.68–0.82).

**TABLE 1:** Emergency department (ED) visits per 100,000 persons, by race/ethnicity — 13 states,* October 1–December 31, 2020

Racial/Ethnic groups	No. of all ED visits	No. (%) of COVID-19 ED visits^†^	No. of COVID-19 ED visits per 100,000 population^§^	RR (95% CI)
**All**	**5,794,050**	282,220 (4.9)	**380**	**—**
Hispanic	759,382	59,204 (7.8)	588	1.77 (1.75–1.78)
AI/AN, non-Hispanic	55,128	3,739 (6.8)	570	1.71 (1.66–1.77)
A/PI, non-Hispanic	125,043	10,788 (8.6)	234	0.70 (0.69–0.72)
Black, non-Hispanic	1,159,086	42,277 (3.6)	463	1.39 (1.38–1.40)
White, non-Hispanic	3,695,411	166,212 (4.5)	333	Referent

**Abbreviations:** AI/AN = American Indian or Alaska Native; A/PI = Asian or Pacific Islander; RR = rate ratio; CI = confidence interval.

* Connecticut, Illinois, Maryland, Massachusetts, Michigan, New Mexico, Nevada, Oregon, Utah, Vermont, Virginia, Washington, and Wisconsin.

^†^ ED visits for COVID-19 are defined as ED visits with any of the following: *International Classification of Diseases, Tenth Revision* (ICD-10) codes U07.1 or J12.82 or Systematized Nomenclature of Medicine (SNOMED) codes 840539006, 840544004, or 840533007.

^§^ Race/ethnicity–specific crude visit rates per 100,000 population were calculated using population denominators from the National Center for Health Statistics 2019 bridged-race postcensal population estimates (https://www.cdc.gov/nchs/nvss/bridged_race.htm); 38,199 (13.5%) ED visits with patient ethnicity data missing or visits with patient ethnicity identified as non-Hispanic/Latino but missing patient race data were not included in this analysis. Patient race or patient ethnicity categorized as “unknown,” “not categorized,” and “refused to answer” are considered missing.

**TABLE 2:** Emergency department (ED) visits per 100,000 persons, by age group and race/ethnicity — 13 states,* October 1–December 31, 2020

Age group, yrs and race/ethnicity	No. of all ED visits	No. (%) of ED visits for COVID-19^†^	COVID-19 ED visits per 100,000 population^§^	RR (95% CI)
**All ages**	**5,794,050**	**282,220 (4.9)**	**380**	**—**
**0–17**				
**All**	**573,105**	**10,049 (1.8)**	**62**	**—**
Hispanic	124,665	3,602 (2.9)	110	2.63 (2.51**–**2.75)
AI/AN, non-Hispanic	5,966	125 (2.1)	76	1.80 (1.51**–**2.15)
A/PI, non-Hispanic	13,890	337 (2.4)	34	0.82 (0.73**–**0.92)
Black, non-Hispanic	114,400	1,986 (1.7)	86	2.04 (1.94**–**2.16)
White, non-Hispanic	314,184	3,999 (1.3)	42	Referent
**18**–**29**				
**All**	**1,001,194**	**28,198 (2.8)**	**231**	**—**
Hispanic	178,845	8,657 (4.8)	431	2.64 (2.56**–**2.71)
AI/AN, non-Hispanic	9,803	425 (4.3)	345	2.11 (1.91**–**2.32)
A/PI, non-Hispanic	20,715	960 (4.6)	110	0.68 (0.63**–**0.72)
Black, non-Hispanic	270,926	5,984 (2.2)	345	2.11 (2.05**–**2.18)
White, non-Hispanic	520,905	12,172 (2.3)	163	Referent
**30**–**44**				
**All**	**1,228,221**	**49,760 (4.1)**	**343**	**—**
Hispanic	193,951	14,933 (7.7)	669	2.77 (2.71**–**2.83)
AI/AN, non-Hispanic	15,167	970 (6.4)	742	3.07 (2.88**–**3.27)
A/PI, non-Hispanic	29,106	2,132 (7.3)	183	0.76 (0.73**–**0.79)
Black, non-Hispanic	295,671	9,507 (3.2)	529	2.19 (2.14**–**2.24)
White, non-Hispanic	694,326	22,218 (3.2)	242	Referent
**45**–**64**				
**All**	**1,525,724**	**91,806 (6.0)**	**480**	**—**
Hispanic	176,102	20,730 (11.8)	1,086	2.92 (2.88**–**2.97)
AI/AN, non-Hispanic	15,996	1,333 (8.3)	844	2.27 (2.15**–**2.40)
A/PI, non-Hispanic	31,620	4,040 (12.8)	376	1.01 (0.98**–**1.04)
Black, non-Hispanic	310,487	14,459 (4.7)	658	1.77 (1.74**–**1.80)
White, non-Hispanic	991,519	51,244 (5.2)	372	Referent
**65**–**74**				
**All**	**682,578**	**46,618 (6.8)**	**646**	**—**
Hispanic	47,429	6,435 (13.6)	1,577	2.83 (2.76**–**2.91)
AI/AN, non-Hispanic	4,967	517 (10.4)	1,025	1.84 (1.69**–**2.01)
A/PI, non-Hispanic	14,302	1,781 (12.5)	562	1.01 (0.96**–**1.06)
Black, non-Hispanic	96,551	5,766 (6.0)	857	1.54 (1.50**–**1.58)
White, non-Hispanic	519,329	32,119 (6.2)	556	Referent
**≥75**				
**All**	**783,228**	**55,789 (7.1)**	**1102**	**—**
Hispanic	38,390	4,847 (12.6)	2,027	1.91 (1.85**–**1.96)
AI/AN, non-Hispanic	3,229	369 (11.4)	1,302	1.22 (1.11**–**1.36)
A/PI, non-Hispanic	15,410	1,538 (10.0)	781	0.73 (0.70**–**0.77)
Black, non-Hispanic	71,051	4,575 (6.4)	1,097	1.03 (1.00**–**1.06)
White, non-Hispanic	655,148	44,460 (6.8)	1,063	Referent

**Abbreviations:** AI/AN = American Indian or Alaska Native; A/PI = Asian or Pacific Islander; RR = rate ratio; CI = confidence interval.

* Connecticut, Illinois, Maryland, Massachusetts, Michigan, New Mexico, Nevada, Oregon, Utah, Vermont, Virginia, Washington, and Wisconsin.

^†^ ED visits for COVID-19 are defined as ED visits with any of the following: *International Classification of Diseases, Tenth Revision* (ICD-10) codes U07.1 or J12.82 or Systematized Nomenclature of Medicine (SNOMED) codes 840539006, 840544004, or 840533007.

^§^ Race/ethnicity–specific age-stratified visit rates per 100,000 population were calculated using population denominators from the National Center for Health Statistics 2019 bridged-race postcensal population estimates (https://www.cdc.gov/nchs/nvss/bridged_race.htm); 38,199 (13.5%) ED visits with patient ethnicity data missing or visits with patient ethnicity identified as non-Hispanic/Latino but missing patient race data were not included in this analysis. Patient race or patient ethnicity categorized as “unknown,” “not categorized,” and “refused to answer” are considered missing.

Among AI/AN persons, those aged 30–44 years had the highest RR of COVID-19 ED visits compared with other age groups (3.07). The RRs among Hispanic persons aged 45–64 (2.92) and 65–74 years (2.83) and Black persons aged 18–29 (2.11) and 30–44 years (2.19) were higher than the other age-stratified estimates for each respective racial/ethnic group. Among Hispanic, AI/AN, and Black persons, the RR of COVID-19-related ED visits was lowest among those aged ≥75 years compared with other age groups (1.91, 1.22, and 1.03, respectively).

## Discussion

Some racial/ethnic groups, including Hispanic, AI/AN, and Black persons, received ED care for COVID-19 at disproportionately higher rates compared with White persons, with higher disparity observed among persons aged <75 years. These findings are consistent with those of previous studies showing disproportionate COVID-19 incidence, hospitalization, and mortality among these racial/ethnic groups (*1*–*4*). Disparities in ED visits for COVID-19 among Hispanic, AI/AN, and Black persons were observed across nearly all age groups, with higher rates in adults aged 18–74 years and the lowest rates among adults aged ≥75 years. Whereas the disparity was lower in this age group, Hispanic and AI/AN persons aged ≥75 years still visited the ED more often than, and Black persons ≥75 years visited the ED as often as, their White counterparts did.

The racial/ethnic groups that sought ED care for COVID-19 at disproportionately higher rates have also experienced long-standing, systemic inequities that affect their health (*6*). These inequities include limited access to quality health care, lower general health status and access to quality education, and disproportionate representation in essential jobs with less flexibility to work from home or take medical leave (*7*). Racism and discrimination shape these factors that influence health risks; racism, rather than a person’s race or ethnicity, is a key driver of these health inequities (*8*). These types of inequities can increase risks for infection with SARS-CoV-2, the virus that causes COVID-19, and delay medical care, increasing the risk for severe COVID-19 outcomes and the need for emergency care.

Effectively protecting and promoting the health of all persons relies on having data to assess and address health disparities. Continued use of NSSP data for ongoing surveillance of COVID-19–related outcomes can serve as an early signal of health disparities experienced by certain racial/ethnic groups. ED data are available in near real-time, and the ability to stratify these data by race/ethnicity provides one of the fastest ways to identify severe outcomes in population subgroups. However, additional efforts to both improve accuracy and completeness of race/ethnicity data and to collect data on social factors that affect health risks should continue. Facility, provider, and public health efforts to improve collection and reporting of these data could aid in rapidly identifying areas of public health concern and understanding of the underlying causes of disparities.

The findings in this report are subject to at least six limitations. First, COVID-19 ED visit data from the 13 assessed states might differ from such data in other states, which could limit the generalizability of these results. Second, White persons represent a larger percentage of the population in the 13-state subset (66%) compared with the national population distribution (61%), so some racial/ethnic groups have less representation, which limits the numbers of observations available and the subsequent inferences that can be made. Third, COVID-19 ED visit classifications rely on diagnostic codes, which might be used inconsistently across facilities, resulting in misclassification of diagnosis. Fourth, persons seek care in EDs for a variety of reasons, including more severe disease or lack of other health care options, and the reasons that someone sought care in an ED rather than another source are not recorded in NSSP data. Fifth, in NSSP, Native Hawaiian and Other Pacific Islander (NH/PI) and Asian are separate categories. However, these groups were combined in the population estimates used, so these groups were combined into A/PI for this analysis, likely masking previously reported COVID-19 disparities among NH/PI persons (*3*). Race/ethnicity–specific estimates for non-Hispanic persons of multiple and other races were not calculated because population denominators were unavailable. Finally, the race and ethnicity fields were categorized based on terms and codes in each visit record. ED visits using nonstandard race or ethnicity descriptors, or missing race/ethnicity data were not included in this analysis.

The findings from this investigation highlight that Hispanic, AI/AN, and Black persons sought ED care for COVID-19 at higher rates than did White persons. It is important to prioritize prevention resources, management of underlying health conditions, safe school and work conditions, flexible leave policies, and enhanced access to and acceptability of SARS-CoV-2 testing and COVID-19 vaccination services to reach disproportionately affected racial/ethnic groups and reduce the need for emergency care for COVID-19. Efforts such as these are critical to address the drivers of racial/ethnic disparities. ED visits from NSSP are an important data source that can be used for near real-time detection of a variety of health conditions, including COVID-19. Race and ethnicity information in these data allows investigators to better identify areas of inequity in their communities and respond by ensuring equitably accessible preventive services, including COVID-19 vaccination, designed to reach the most affected communities.

**Summary**

**What is already known about this topic?**

Hispanic, American Indian or Alaska Native, and Black persons have higher rates of hospitalization and death attributable to COVID-19 than do White persons.

**What is added by this report?**

Data from 13 states indicate that compared with White persons, Hispanic and American Indian or Alaska Native persons experienced 1.7 times the rate, and Black persons experienced 1.4 times the rate of emergency department care visits for COVID-19 during October–December 2020.

**What are the implications for public health practice?**

Emergency department COVID-19 visit data can provide insight into ongoing areas of racial/ethnic inequity in health status and disease outcomes and can be used to prioritize prevention resources, including COVID-19 vaccination, to reach disproportionately affected groups.

## Footnotes

* ED visits for COVID-19 are defined as ED visits with any of the following: *International Classification of Diseases, Tenth Revision* (ICD-10) codes U07.1 or J12.82 or Systematized Nomenclature of Medicine (SNOMED) codes 840539006, 840544004, or 840533007.

† A total of 38,199 (13.5%) ED visits with patient ethnicity data missing or visits with patient ethnicity identified as non-Hispanic/Latino but missing patient race data were not included in this analysis. Patient race or patient ethnicity categorized as “unknown,” “not categorized,” and “refused to answer” are considered missing.

§ RR of COVID-19 ED visits: (COVID-19 ED visits among persons in the racial or ethnic age group of interest/population of persons in the racial or ethnic age group)/(COVID-19 ED visits among White persons in that age group/population of White persons in that age group).

¶ 45 C.F.R. part 46; 21 C.F.R. part 56; 42 U.S.C. Sect. 241(d), 5 U.S.C. Sect. 552a, 44 U.S.C. Sect. 3501 et seq.
